# Odontological Society of Pennsylvania

**Published:** 1895-06

**Authors:** 

**Affiliations:** Odontological Society of Pennsylvania


### ODONTOLOGICAL SOCIETY OF PENNSYLVANIA.

The regular monthly meeting of this Society was held January 12, 1895, President Dr. C. N. Peirce in the chair.

At the conclusion of routine business, Dr. Joseph Head read a paper entitled "Plastics as a Bar to Bacteria."

(For Dr. Head's paper, see page 349.)

DISCUSSION.

*Dr. Brubaker.*—I hardly think I have anything to say on this subject. The first fact to be considered is, Does the fluid in the tooth that is filled pass through the filling to the interior of the tooth? And, secondly, assuming the fluid does pass through the tooth, is it likely that bacteria will follow the fluid? Might it not be possible that fluids can go through without conducting bacteria. Of course, these bacteria can be readily seen by a sufficiently high-power microscope. I would like to know what power lens the essayist employed to detect these bacteria.

The Pasteur filter is composed of clay that has been burned. We find water will pass through it under very considerable pressure, but after three months continuous use bacteria will be found in enormous numbers on the outside of that clay; but it has been almost impossible ever to find bacteria on the inside of it. And this is true not only of water, but of beef broth and a great many other fluids.

Therefore, the only fact in this paper which I see is, first, the demonstration that fluids pass through the teeth; secondly, the demonstration of bacteria going through with the fluids. The writer states that they have gone through. He does not state what kind of bacteria.

*Dr. Head.*—Single micrococci, two joined in dumb-bell fashion, and large numbers of colonies. Miss Byrnes, the Fellow of Biology of Bryn Mawr, also examined them, and it was she who really had to decide whether bacteria were present, and on the examination of each cone she saw them. We had an immersion lens, magnifying eight hundred diameters.

If there is any microscopist in the company who doubts the presence of bacteria, there are still some unopened cones in the bouillon which I shall be very glad to put at his disposal.

Dr. Smith was called upon, but did not respond.

*Dr. Faught.*—I have nothing to say in addition to the scientific character of the paper, but desire to express my appreciation. Such a subject requires going over very carefully in its details before one can speak with any accuracy upon it, and it is valueless unless it is accurate. In looking it over I do not, at the first glance, see anything that could be questioned, unless it was the question of sterilization, and I would not like to raise that, because the method employed seems to be thorough. We have not only the testimony of Dr. Head, but also the additional testimony of learned authority, which points to its having been carefully and accurately done. This is a paper that would bear discussion at a future date more than at the present time, after some one had had an opportunity of verifying the conés enclosed.

*Dr. James Truman.*—This paper seems to me a mere question of fact, and one that we cannot well discuss. I cannot understand how micro-organisms can enter into cement fillings, or fillings of tin, gold, or amalgam, and yet those fillings preserve the teeth. That is the only point that seems to me objectionable in these experiments. I think it requires further work. If micro-organisms, if pathogenic germs, enter through fillings, how is it possible that fillings of that character preserve the walls. I have no doubt at all that these enter some fillings; but I cannot comprehend how this is possible with cohesive gold thoroughly packed. If that filling is flush with the walls, it must be preservative. The question is whether it is possible to absolutely make a malleted cohesive gold filling flush with the wall so it will prevent ingress of micro-organisms. I do not regard it as impossible, but it is very difficult.

But if micro-organisms penetrate into the plastics, as they seem to have done in the experiments of Dr. Head, the question arises, How is it that fillings preserve the teeth? But this is a question foreign to the paper. He has simply demonstrated a single but a very important point, that micro-organisms can enter into a plastic filling. Now, we know that in the oral cavity there are many kinds of germs, pathogenic and non-pathogenic. Whether a certain kind of micro-organisms can enter into fillings of this character while they prevent the ingress of others that are destructive to the tooth-structure needs further investigation.

*Dr. Head.*—There is one point apropos of what Dr. Truman has just said. The bouillon was sterilized first, and then inoculated with the decayed tooth substance. So it would seem that a large part of the germs found were the germs of tooth decay.

*Dr. Peirce.*—As Dr. Head has two or three cones yet unopened, I would like to suggest that one be given to Dr. Truman and one to Dr. Brubaker, and so enable them to report on their contents at some future meeting.

Dr. Broomell then read a paper in defence of an article which was criticised by Dr. William H. Trueman in the INTERNATIONAL DENTAL JOURNAL.

Adjourned.

JOSEPH HEAD, M.D., D.D.S.,
*Editor Odontological Society of Pennsylvania.*

